# Systemic inflammatory response syndrome in a patient diagnosed with high grade inflammatory triple negative breast cancer: a case report of a potentially rare paraneoplastic syndrome

**DOI:** 10.1186/s40164-016-0045-2

**Published:** 2016-06-22

**Authors:** Piers R. Boshier, Rosie Sayers, Dimitri J. Hadjiminas, Charles Mackworth-Young, Susan Cleator, Daniel R. Leff

**Affiliations:** 1 Department of Surgery and Cancer, Imperial College London, London, UK; 2 Department of Breast Surgery, Imperial College NHS Healthcare Trust, Charing Cross Hospital, London, UK; 3 Department of Rheumatology, Imperial College NHS Healthcare Trust, Charing Cross Hospital, London, UK; 4 Department of Oncology, Imperial College NHS Healthcare Trust, Charing Cross Hospital, London, UK

**Keywords:** Inflammatory breast cancer, Systemic inflammatory response syndrome, Paraneoplastic syndrome, Case report

## Abstract

**Background:**

Inflammatory breast cancer is a complex pathological entity associated with poor outcomes. This loco-regional disease is characterised by a rapid clinical course in the presence breast erythema and infiltration of dermal lymphatics by tumours cells. Herein we describe a case of inflammatory breast cancer with a rare presentation and disease course defined by a profound systemic inflammatory response in the absence of an infective cause.

**Case presentation:**

The patient presented with pyrexia and malaise following a recent tissue diagnosis of inflammatory breast cancer. At the time of admission the patient demonstrated clinical features of the systemic inflammatory response syndrome (SIRS) in the presence of a negative septic screen. Her condition deteriorated despite systemic broad spectrum intravenous antibiotics and she underwent surgical debulking of a 180 × 135 × 100 mm (821 g) primary tumour composed of oedematous, friable and haemorrhagic tissue (pT4,N1a,M0; oestrogen/progesterone/HER-2 receptor negative). Following surgery, the clinical picture dramatically improved with cessation of SIRS and normalisation of inflammatory markers. After 4 weeks the patient required readmission to hospital due to recurrent SIRS and negative septic screen. The patient received treatment with systemic chemotherapy showing transient clinical improvement and suppression of SIRS. Despite on going chemotherapy, systemic antibiotics and a trial of steroid therapy the patient died 5 months after her initial presentation to hospital. At the time of death she demonstrated persistent SIRS with elevated inflammatory markers.

**Conclusion:**

This is the first case report of inflammatory breath cancer associated with SIRS in the absence of clinically confirmed infection. Important learning points highlighted by this case are: (a) recognition of the diagnostic and therapeutic uncertainties that still exist in the context of inflammatory breast cancer; (b) appreciation of the potential paraneoplastic systemic inflammatory manifestations of this disease, and finally; (c) the importance a multidisciplinary and multimodal approach to treatment.

## Background

Inflammatory breast cancer (IBC) is a complex clinicopathological entity that is associated with a poor prognosis [1]. Consensus guidelines for the diagnosis of IBC describe a locoregional disease characterised by rapid onset breast erythema, oedema and/or peau d’orange with or without an underlying palpable mass [1]. Whilst localised skin warmth may be present, a systemic inflammatory response is not a recognised feature of this disease and if present is likely to suggest an alternative diagnosis.

The systemic inflammatory response syndrome (SIRS) is a multifaceted pathophysiological response to a range of noxious stimuli, whose characteristics were first defined in 1992 [2]. The link between inflammation and cancer has long been established [3] and is associated with readily detectable clinical and biochemical changes [4]. The mechanism by which cancer induces both local and systemic inflammatory responses is yet to be fully elucidated, although it is thought to involve complex interactions between cancerous cells and adjacent stroma leading to tissue damage and initiation of an acute phase response [3].

Traditionally the *‘inflammatory’* response observed in IBC is attributed to the infiltration of dermal lymphatics by tumour cells, which in turn causes congestion. A potent inflammatory milieu has nevertheless been observed in the context of IBC [5, 6]. Furthermore, raised serum inflammatory markers are recognised in the serum of up to one-third of patients with grade I-III breast cancer [7]. The underlying mechanism clinical significance of SIRS in the context of a cancer diagnosis is however yet to be determined.

Herein, we present an unusual case of inflammatory breast cancer associated with a systemic inflammatory response in the absence of clinical evidence of infection.

## Case presentation

A 48 year old pre-menopausal Jamaican female, recently diagnosed with IBC affecting the left breast, was admitted with a 5 day history of pyrexia and general malaise. Her co-morbidities included hypertension and sarcoidosis treated with antihypertensives and low dose oral prednisolone respectively. There was no relevant family or travel history and no history of recent illness.

An ultrasound scan performed at the time of diagnosis, 6 weeks prior to admission, showed a 41 mm ill-defined mass in the upper outer quadrant of the left breast with bilateral axillary nodal enlargement. Computed tomography (CT) imaging at the time of diagnosis revealed no evidence of solid organ metastasis within the chest, abdomen or pelvis. Core biopsy of the lesion confirmed a pleomorphic, poorly differentiated triple negative (estrogen/progesterone/human epidermal growth factor (HER2) receptor) grade III invasive ductal carcinoma.

At the time of admission, clinical examination revealed a large (~5 × 10 cm) left upper outer quadrant breast mass with associated erythema and induration of the surrounding tissue and palpable lymphadenopathy of within the axilla. The diagnostic criteria for systemic inflammatory response syndrome (SIRS) were fulfilled [heart rate 120; respiratory rate 26; temperature 38.7 °C; white blood cell count 16.9 × 10^9^/L (neutrophils 14.9 × 10^9^/L)] [2]. Biochemical markers of acute inflammation were observed to be markedly deranged (C-reactive protein (CRP) 385 mg/L; erythrocyte sedimentation rate >100 mm/h; ferritin 1044 μg/L; transferrin 0.6 g/L; albumin 16 g/L). Blood cultures at the time of admission, before initiation of antibiotic therapy, demonstrated no bacterial growth. Likewise an ultrasound guided aspirate of tissue fluid contained within the mass yielded no evidence of bacterial infection.

Despite treatment with antibiotics (Cefuroxime and Metronidazole), 48 h after admission worsening SIRS was observed (heart rate 114; respiratory rate 24; Temperature 39.0 °C; White blood cell count 20.0 × 10^9^). A CT scan 72 h after admission demonstrated an increase in size of the primary breast lesion (75 × 94 vs. 38 × 80 mm), features in keeping with pectoralis major muscle invasion and associated ipsilateral lymphadenopathy and cervical chain adenopathy. A left upper lobe pulmonary nodule (9 mm) and para-aortic lymph node (8 mm) suspicious of metastatic spread were also noted in addition to small bilateral pleural and pericardial effusions. Subsequent transthoracic echocardiogram and CT head revealed no additional abnormalities or likely source of infection. Serum virology and autoimmune screens were negative as were repeat blood and urine cultures.

Over the subsequent week she experienced continued clinical deterioration with anaemia (haemoglobin; 87–65 g/L) and coagulopathy (fibrinogen 5.65 g/L; prothrombin time 17.2 s; activate partial thromboplastin time 40.6 s) that required transfusion of packed red cells (6 units) and fresh frozen plasma (3 units). There was no evidence of active bleeding and haemoglobin electrophoresis was normal. A blood film showed signs of anaemia with significant red cell hypochromia, anisopoikilocytosis, hypersegmentated neutrophils and thrombocytosis with platelet clumping.

**Fig. 1:**
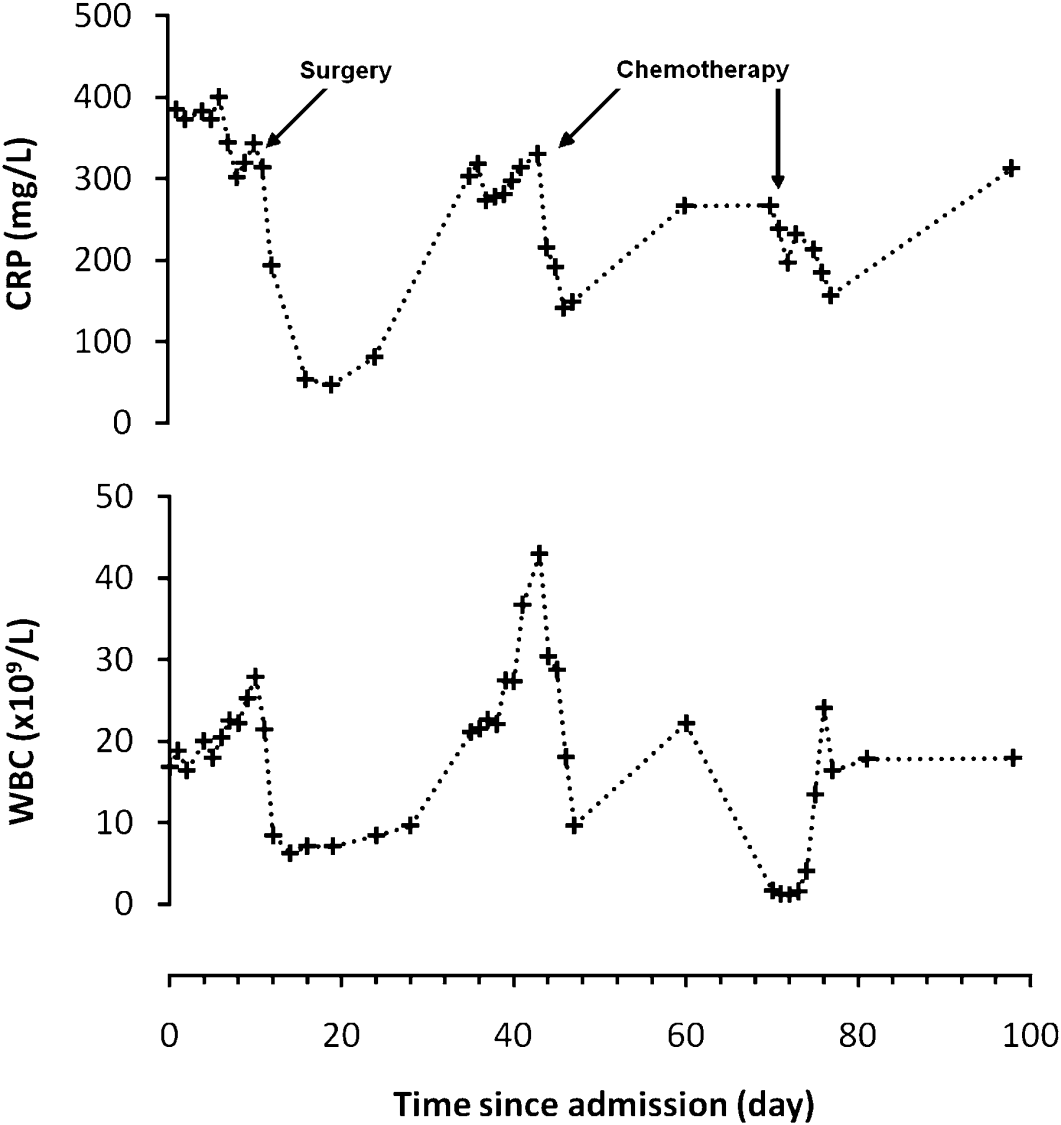
C-reactive protein *CRP* and white blood cell *WBC* trends from time of acute admission to hospital

**Fig. 2:**
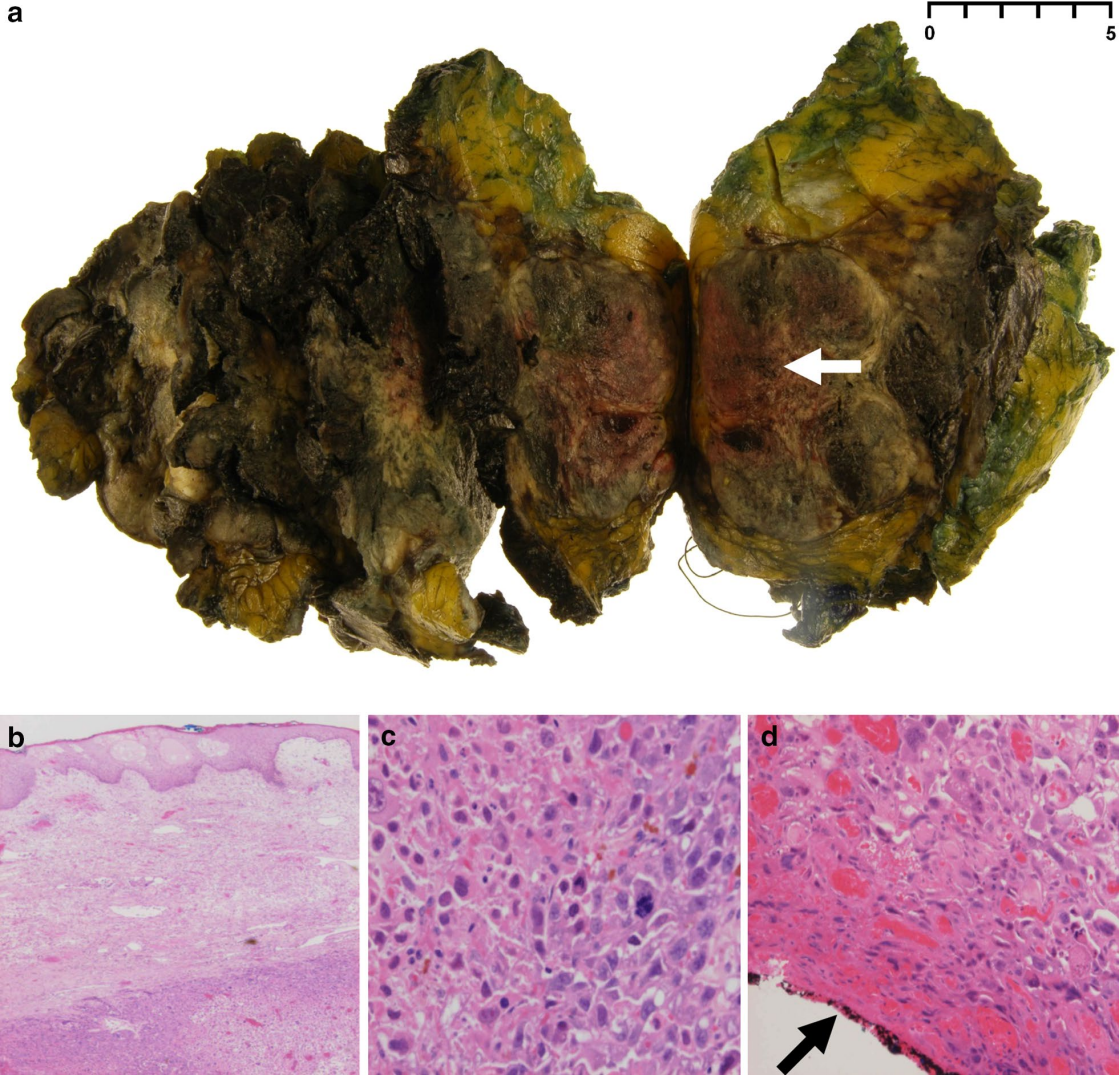
Macro- and microscopic tumour appearance. **a** Macroscopic image of excised breath tissue with section through tumour (*white arrow*). Microscopic images demonstrating: **b** tumour cells and lymphovascular invasion of the superficial epidermis; **c** high magnification image of tumour cells, and; **d** tumour involvement of the deep excision margin (*black arrow*)

The patients condition deteriorated over the course of 9 days with no evidence of a response to broad spectrum antibiotic therapy (including: Vancomycin; Ciprofloxacin; Tazocin, and; Meropenem) (Fig. 1) and persistently worsening SIRS and episodic hypotension without objective evidence of an infective source. Formal nutritional assessment revealed good nutritional intake and no specific deficiency. Consequently the multidisciplinary team advised emergency excision of the left breast tumour mass on the assumption that the SIRS might represent a response to necrotic inflammatory cancer. At the time of surgery a 180 × 135 × 100 mm (821 g) diffusely oedematous, friable and haemorrhagic tissue mass was excised (Fig. 2). A delayed primary closure of the wound was performed on the seventh postoperative day with Yates drain left in situ. Histology of the resected specimen confirmed grade III pleomorphic carcinoma (>100 mm) with adjacent high grade ductal carcinoma in situ, lymphovascular invasion and extracapsular spread and significant necrosis (pT4,N1a,Mx; oestrogen/progesterone/HER-2 receptor negative, E-cadherin/cytokeratin-7/p53/p63 positive) (Fig. 2). Radial margins were clear (>2 mm) but the deep resection margin was involved.

Following surgery the patient’s clinical picture dramatically improved with a cessation of SIRS and normalisation of inflammatory markers. At the time of hospital discharge on (postoperative day 8), the patient was clinically well. A multidisciplinary team decision for the continuation of therapy as an outpatient had been agreed. Approximately 4 weeks later she represented with recurrent SIRS and a left breast seroma which was formally drained (1.5L haemoserous fluid) and which was negative on culture. Critically, no infective source could be identified (blood, urine, seroma aspirate culture were negative). Positron emission tomography–CT performed soon after readmission demonstrated a recurrent mass (32 × 77 mm) at the site of the original tumour. In addition there was progressive bilateral lymphadenopathy including mediastinal nodes and bilateral intrapulmonary masses with the largest present in the superior segment of the lingual measuring (22 × 40 mm). There was heterogeneity of the hepatic fludeoxyglucose uptake with increase skeletal activity, indicative of metastatic disease. Chemotherapy (FEC regimen; fluorouracil, epirubicin and cyclophosphamide) was started 6 weeks after her initial emergency presentation. The first cycle of chemotherapy was well tolerated and associated with resolution of SIRS and improvement in inflammatory markers. Whilst receiving the second cycle of chemotherapy as an outpatient, she developed neutropenia and pyrexias (culture negative) for which she received a further course of broad spectrum antibiotics and epidermal growth factor.

She received a third cycle of FEC chemotherapy following which a repeat CT scan showed an increase in size of the primary tumour, multiple pulmonary metastasis with extensive nodal disease.

A subsequent aspiration of the breast cavity grew staphylococcus aureus, pseudomonas and enterococcus for which she was prescribed Linezolid and Meropenem but without a clinical response.

The Oncology team discussed whether second line taxane chemotherapy was appropriate, but this was withheld because the patient’s condition deteriorated rapidly. The albumin at this point was 13 g/L.

Despite no impedance to the investigation, diagnosis and management of this patient during her care she died 5 months after her initial presentation to hospital. At the time of death she demonstrated persistent SIRS with elevated markers of inflammation (white blood cell count 47.6 × 10^9^/L (Neutrophils 44.5 × 10^9^/L); CRP 332 mg/L).

## Discussion

This is the first case report of IBC associated with SIRS in the absence of clinically defined infection in the English medical literature. Important learning points highlighted by this case are as follows: (a) recognition of the diagnostic and therapeutic uncertainties that still exist in the context of IBC; (b) appreciation of the potential para-neoplastic systemic inflammatory manifestations of this disease, and; (c) the importance a multidisciplinary and multimodal approach to treatment.

One feature of this case that is particularly remarkable was the magnitude of the observed systemic inflammatory response. A systematic review of the literature revealed no previously published report of an equivalent systematic inflammatory response to IBC. Several case reports have described: fever [8]; elevated erythrocyte sedimentation rate [9]; elevated CRP, and; raised white blood cell count and in the presence of a negative septic screen as well as pure red cell aplasia [10] in patients diagnosed with IBC. Molecular profiling has sought to provide further insight into the pathogenesis of IBC [5]. Studies have found evidence for the up-regulation of several genes associated with inflammatory signalling pathways in IBC cells compared to non-IBC cells. Biѐche et al., reported up regulation of interleukin (IL) 6 and genes encoding the CCL3/MIP1A and CCL5/RANTES [5]. Likewise NF-κB, cyclooxygenase family of enzymes and JAK/STAT signalling, all responsible for a range of effects including propagation of the inflammatory and immune response, are constitutively active in IBC [6]. Of note, the genetic expression of other important inflammatory mediators, tumour necrosis factor alpha (TNFα), Interferon, IL-1, IL-8 and IL-10 was similar in IBC and non-IBC cells [5]. Significantly increased inflammatory cytokine (TNFα, IL-8, IL-10 and CCL2/MCP-1) secretion was however observed in CD14+ monocytes isolated from IBC compared to non-IBC patients [11]. Finally, the acute phase protein CRP has been correlated to a more aggressive disease phonotype and poorer prognosis in breast cancers patients [12]. Sphingosine-1-phosphate, a potent inflammatory mediator, has recently been reported to up-regulate the expression of CRP in breast cells [13]. Whilst these findings support the premise of an inflammatory basis for IBC their wider implications for a systemic inflammatory response are yet to be fully elucidated.

The patient fulfilled all of the clinical criteria for the diagnosis of SIRS as defined by the American College of Chest Physicians/Society of Critical Care Medicine [2]. An additional feature of this case was observed derangement of red blood cell count and clotting function that required treatment with blood products. The Third International Consensus Definitions for Sepsis and Septic Shock defines sepsis as a “life-threatening organ dysfunction caused by a dysregulated host response to infection” [14]. Critically after extensive and repeated investigation at the time of presentation and during the initial period of treatment no evidence was found for an infective origin for this response. Whilst positive bacterial cultures were eventually grown from the surgical wound cavity, isolated organisms are reported amongst the most common causes of hospital acquired wound infection [15, 16]. As such the eventual presence of these bacteria in the wound is most in keeping with nosocomial infection. Numerous factors, including previous surgery, chemotherapy and general cachexia would strongly predispose this patient to such infection.

The unusual clinical course exhibited by this patient proved challenging both in terms of establishing an underlying mechanism and determination of appropriate management. Existing criteria for a diagnosis of neoplastic fever are not met in this specific case [17, 18]. Measurement of plasma procalcitonin is an alternative method of differentiating sepsis from paraneoplastic fever that may have help direct this patients management. Procalcitonin, a pre-cursor of the hormone calcitonin, is found in significantly higher levels in patients with sepsis compared to febrile patients with no documented evidence of infection [18]. This investigation is however not widely available and the merits of use are still debated. Lymph node biopsy at the time of presentation may also have help determine if another cause of lymphadenopathy, other than tumour metastasis, was responsible for the patients clinical picture.

A second remarkable characteristic of this case was the dramatic effects of surgical excision and subsequent chemotherapy on suppression of the systemic inflammatory response, albeit only temporary. Furthermore, the transient nature of the response to surgical excision only served to emphasise the extremely aggressive nature of the underlying disease process. Consensus guidelines for the management of IBC published by Dawood et al. and the National Comprehensive Cancer Network, recommend primary systemic chemotherapy as the first line treatment with aim of down-staging the disease allowing the possibility for definitive surgery [1, 19]. In this specific case, however, due to the profound clinical deterioration and in the absence of a confirmed source of sepsis a multidisciplinary team decision was taken to excise the tumour mass for fear that the patient may not survive without surgical resection. Ultimately the observed SIRS response was only transiently suppressed by surgical resection and cycles of systemic chemotherapy.

The presence of a prior diagnosis of sarcoidosis, an immunologically mediated disease, may have contributed to the observed systemic inflammatory response. Whilst the pathophysiology of sarcoidosis is itself incompletely understood, it is recognised that a low activation threshold and dysregulation of the immune system are important for its development and progression [20]. A systematic review of the literature reveals no previous published reports of IBC in the presence of sarcoidosis. Notwithstanding, it may be hypothesised that in the context of existing immune dysfunction, the local and systemic inflammatory effects of IBC can be significantly augmented.

## Conclusion

This case report represents the first description of a potential paraneoplastic systemic inflammatory response in the context of inflammatory breast cancer. This report contributes to the existing published literature that seeks to characterise and define the underlying link between the inflammatory process and IBC. It also serves as a reminder of the aggressive and unpredictable nature of this disease and offers new insight into the challenges faced by clinicians charged with its management.

## Abbreviations

CCL2/MCP-1: chemokine (C–C motif) ligand 2/monocyte chemotactic protein 1
CCL3/MIP1A: chemokine (C–C motif) ligand 3/macrophage inflammatory protein 1-alpha
CCL5/RANTES: chemokine (C–C motif) ligand 5/regulated on activation, normal T cell expressed and secreted
CD14+: cluster of differentiation 14+
CRP: C-reactive protein
CT: computer tomography
FEC: fluorouracil, epirubicin and cyclophosphamide
HER2: human epidermal growth factor receptor 2
IBC: inflammatory breast cancer
IL: interleukin
JAK/STAT: janus kinase/signal transducer and activator of transcription
NF-κB: nuclear factor kappa-light-chain-enhancer of activated B cells
SIRS: systemic inflammatory response syndrome
TNFα: tumour necrosis factor alpha
